# Rapid-Testing Technology and Systems Improvement for the Elimination of Congenital Syphilis in Haiti: Overcoming the “Technology to Systems Gap”

**DOI:** 10.1155/2013/247901

**Published:** 2013-12-22

**Authors:** Linda Severe, Daphne Benoit, Xi K. Zhou, Jean W. Pape, Rosanna W. Peeling, Daniel W. Fitzgerald, Kedar S. Mate

**Affiliations:** ^1^GHESKIO Centres, Port-Au-Prince, Haiti; ^2^Department of Public Health, Weill Cornell Medical Center, New York, NY, USA; ^3^Global Health Center, Weill Cornell Medical Center, New York, NY, USA; ^4^London School of Tropical Medicine, London, UK; ^5^Department of Medicine, Weill Cornell Medical Center, 525 East 68th Street, P.O. Box 130, New York, NY 10065, USA; ^6^Institute for Healthcare Improvement, Cambridge, MA, USA

## Abstract

*Background*. Despite the availability of rapid diagnostic tests and inexpensive treatment for pregnant women, maternal-child syphilis transmission remains a leading cause of perinatal morbidity and mortality in developing countries. In Haiti, more than 3000 babies are born with congenital syphilis annually. *Methods and Findings*. From 2007 to 2011, we used a sequential time series, multi-intervention study design in fourteen clinics throughout Haiti to improve syphilis testing and treatment in pregnancy. The two primary interventions were the introduction of a rapid point-of-care syphilis test and systems strengthening based on quality improvement (QI) methods. Syphilis testing increased from 91.5% prediagnostic test to 95.9% after (*P* < 0.001) and further increased to 96.8% (*P* < 0.001) after the QI intervention. Despite high rates of testing across all time periods, syphilis treatment lagged behind and only increased from 70.3% to 74.7% after the introduction of rapid tests (*P* = 0.27), but it improved significantly from 70.2% to 84.3% (*P* < 0.001) after the systems strengthening QI intervention. *Conclusion*. Both point-of-care diagnostic testing and health systems-based quality improvement interventions can improve the delivery of specific evidence-based healthcare interventions to prevent congenital syphilis at scale in Haiti. Improved treatment rates for syphilis were seen only after the use of systems-based quality improvement approaches.

## 1. Introduction 

Congenitally acquired syphilis remains a leading cause of perinatal morbidity and mortality in developing countries with an estimated 1.5 million annually affected pregnancies worldwide [1]. In Haiti, studies document that more than 3,000 babies are born with congenital syphilis each year [2]. More than half of these result in still-births or perinatal death [3, 4].

Syphilis is easily detected during antenatal care using rapid test technology for point-of-care diagnosis [5, 6]. Same day treatment can be provided with a single shot of penicillin preventing more than 90% of congenital transmission [3]. Point-of-care diagnosis and same-day treatment can prevent high rates of lost to follow up that occur when treatment is provided at a second visit. Studies in Haiti and Sub-Saharan Africa demonstrate that when treatment is provided at a second visit, nearly fifty percent of women who test positive for syphilis never receive treatment [7]. Point-of-care diagnosis and same-day treatment are inexpensive, and financial analyses in Haiti and elsewhere have demonstrated their cost-effectiveness [8, 9]. Remarkably, same-day care has been known for more than 25 years, yet reliable national implementation of this practice remains elusive.

Recognizing the cost-effectiveness of point-of-care diagnosis and same-day treatment of syphilis in pregnant women, the Haitian Ministry of Health has established a national goal of eliminating congenital syphilis by 2015 [2]. Here, we report on the introduction of rapid test technology and health systems improvement intervention at national scale in Haiti to improve syphilis care and treatment for pregnant women. Their sequential implementation allowed us to measure their independent effects on syphilis case detection and treatment.

## 2. Methods

### 2.1. Ethics Statement

Approval for this study was obtained from the ethics review boards of both the GHESKIO Centres and Weill Cornell Medical College.

### 2.2. Study Sites

Fourteen geographically distributed clinics throughout Haiti were selected for participation in this study. We selected larger clinics that were supported by the US Government's PEFPAR program to provide PMTCT services for pregnant women and that were supervised by staff from the GHEKSIO Centres, a Haitian nongovernmental organization based in Port-au-Prince. We describe the facility-based characteristics of these sites including public versus private, rural versus urban, and geographic location.

### 2.3. Study Design

Rapid syphilis testing technology and a systems-based improvement intervention were introduced sequentially to these fourteen PEPFAR-supported clinics over 51 months from 2007 to 2011. In the first phase of the study, a syphilis point-of-care test (Standard Diagnostics BIOLINE, Syphilis 3.0) was introduced to all clinical study sites between January 2008 and April 2009. This was part of a national program to incorporate point-of-care syphilis testing at all PEPFAR sites in the country that were testing pregnant women for HIV. In the second phase, a systems-improvement intervention was introduced to all 14 study sites simultaneously in October 2010. A time series analysis was utilized to understand the effects of these interventions occurring sequentially over the study period.

### 2.4. Interventions: Point of Care Tests with Traditional Educational Program

In 2008, the Haitian Ministry of Health introduced routine prenatal syphilis screening using a rapid syphilis test as part of a national plan to eliminate congenital syphilis. Clinic staff attended a two-day lecture-based training on the epidemiology of syphilis, modes of transmission, clinical manifestations, diagnosis, treatment, and prevention of congenital syphilis. Laboratory technicians also attended an additional one-day workshop to learn how to perform the rapid point of care test. SD BIOLINE is a whole blood, immunochromatographic assay for the qualitative detection of antibodies of all isotypes against *Treponema pallidum* (SD Bioline Syphilis 3.0, Kyonggi-Do, republic of Korea). No additional equipment is required and all reagents can be stored at room temperature. Test results were available within 30 minutes at the point of care. Quality assurance of the rapid test was done according to the manufacturer's recommendations. Each rapid test includes a control test “band” to indicate the appearance of a negative test. In addition, GHESKIO runs known positive and known negative serums to assure rapid test validity periodically. Studies from Haiti and other countries show that the test is highly sensitive and specific for the diagnosis of syphilis in pregnant women [10, 11]. In our experience in Haiti, a positive rapid test is also positive by RPR and confirmatory TPHA more than 95% of the time, suggesting that the rapid test is diagnosing active infection in more than 95% of cases.

### 2.5. Systems-Based Quality Improvement

Prior systems strengthening efforts to improve coverage of syphilis care and treatment have focused on laboratory support, supply chain improvements, workforce training, and monitoring and evaluation [12]. In 2010, GHESKIO used a systems-based quality improvement (QI) approach to strengthen prenatal syphilis screening and treatment. The effectiveness of the QI approach to strengthen care delivery systems for tuberculosis, HIV, maternal and child health, and PMTCT has been documented in other resource-poor countries [13–16]. The QI approach used a conceptual framework called the “model for improvement” based on the original work of statistician Deming [17, 18]. The approach uses local facility-based systems improvement teams, composed of facility staff, to select the best evidence-based healthcare interventions, to conduct local gap and root-cause analyses (fishbone Ishikawa diagrams) to diagnose systems failures, and to use the plan-do-study-act (PDSA) rapid cycle change approach to improve failing systems [17].

The QI approach addressed implementation failures at each clinic using three techniques taught to clinic staff during two workshops.

1. *Forming Multidisciplinary, Facility-Based Systems-Improvement Teams*. These teams, established at the beginning of the intervention period, were composed of the facility manager, at least one nurse, the data clerk, and other interested facility members. Each participating site formed a systems-improvement team and the size of these teams varied based on the size of the clinic and availability of staff. All teams established clear, facility-based improvement goals in each clinical area, used root-cause analysis to understand system barriers and process mapping to understand patient flow and bottlenecks in clinical pathways. They were asked to meet on a twice-monthly basis to oversee the QI activities.
2. *Conducting Local Process Indicator Analysis*. QI experts from GHESKIO (2 of them) assisted the facility-based teams to use their own local register data to conduct facility-specific data analysis using Pareto and basic run-chart analysis [19]. Such Pareto and run-chart analyses allowed facility teams to visually depict service delivery gaps that were present in their day-to-day work. This analysis was completed on a monthly basis for all clinics.
3. *Using the Model for Improvement Systems Change Strategy*. Facility-based QI teams used a problem-solving strategy based on the conceptual framework offered by the model for improvement to implement solutions to local barriers [17]. Job aids, distribution of guidelines and new protocols, training materials, changes to clinic flow, and other specific systems improvement tools were employed to solve specific systems failures or to reinforce the reliable performance of a desired clinic behavior. The tool or technique which would be successful for each individual clinic was not predicted at the outset. Each PDSA cycle culminated in a decision about whether to adopt, adapt, or abandon a specific systems change based on whether it had produced an improved service. Each clinic was encouraged to complete one PDSA cycle each month during the intervention period.

We documented the primary service delivery failures and the corresponding specific solutions that were tested and implemented by these facility-based teams. These data were gathered during the two QI training sessions through two techniques: focus group interviewing and presentations by each facility involved with the initiative on key systems barriers, solutions tested, and results obtained.

In all cases, partner notification was done by sending an invitation card through the patient/pregnant woman. Data on uptake of this intervention were not tracked as part of this study; however, GHESKIO and its partners made every effort to urge pregnant women to communicate their status to their partners and would link them to testing and treatment as soon as they presented for care.

### 2.6. Study Endpoints

The study focused on two clinical measures: the proportion of all pregnant women tested for syphilis and receiving their results, and the proportion of those women testing positive who were subsequently treated for syphilis during the antenatal period.

### 2.7. Data Collection and Analysis

Data were obtained from the web-based MESI.HT health information system of the Haitian Ministry of Health. Data were analyzed over a 51-month time period, divided into four phases: prerapid test (March 2007–March 2009), postrapid test initiation (April 2009–December 2009), prequality improvement (January 2010–September 2010), and postquality improvement initiation (October 2010–June 2011). We also examined the effect of the January 2010 earthquake on testing and treatment rates. Logistic regression was used to compare testing and treatment rates in all four study periods. Differences in the testing and treatment rates between periods were further examined using simultaneous tests for general linear hypotheses. *P* values were controlled for multiple comparisons using the Bonferroni method. Differences in clinic specific testing and treatment rates in the four study periods were also examined for all clinics in aggregate, for each individual clinic, and for subgroups of clinics (public, private, urban and rural). This analysis allowed us to see whether each intervention was associated with a significant increase or decrease in the outcome variables across all participating clinics in each individual clinic and in each type of clinic. For clinics where rates were close to 100%, Fisher's exact test was used to compare rates.

Using population estimates from the Ministry of Health, rates of congenital syphilis, and the results of the logistic regression, we estimated the effects of these interventions if they were scaled up country wide. Our estimation is based on a previously described model by Schackman et al. [9] using the following assumptions: 320,000 pregnancies in Haiti annually, a 4% syphilis positivity rate country wide, and a 43.8% serious birth complication (stillbirth or neonatal death) rate for untreated mothers and a 1.1% complication rate for treated mothers [3].

## 3. Results

Fourteen clinical sites participated in the study. Twelve sites were characterized as hospitals (bed size ranged between 10 and 150) and two as clinics. The sites were evenly split between publicly managed (*n* = 7) and privately operated by nongovernmental organizations (NGOs). Eleven (78%) were situated in urban settings and three (22%) were located in rural settings. The average population in the catchment area for the urban health facilities was 410,000 individuals (range = 80,000–1.6 million). Average catchment areas for the rural facilities was 55,000 (range = 32,000–79,000). All facilities received the rapid syphilis test, and all facilities received the two systems-based QI workshops. Under the systems-based QI intervention, all facilities composed a multidisciplinary team and all conducted at least one PDSA cycle during the study period.

### 3.1. Syphilis Testing

During the baseline period, the overall syphilis testing rate was 91.5% (Table 1). During the nine months after the introduction of the rapid syphilis test, the syphilis testing rate increased to 95.9% (*P* < 0.001). After the January 2010 earthquake, the testing rate remained similarly at 95.8%. After the QI intervention, further small yet significant increase in testing rate to 96.8% was observed (*P* < 0.001).

Despite the overall improvement in testing rates following the interventions, substantial variation in subgroups of clinics was observed. After the introduction of the rapid test, syphilis testing rates in public clinics increased from 87.0% to 96.1% (*P* < 0.001) whereas private clinics decreased from 98.1% to 95.8% (*P* < 0.001). After the QI intervention, public clinics improved significantly (93.2% to 95.5%, *P* < 0.001) while private clinics were not significantly different (99.4% to 99.2%).

### 3.2. Syphilis Treatment

**Figure 1:**
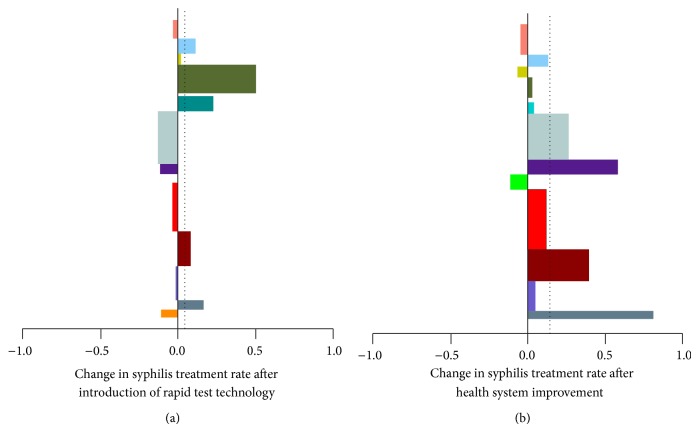
Change in syphilis treatment rate after the rapid syphilis test (a) and the introduction of quality improvement. (b) Each bar represents one of the fourteen participating clinics and the direction represents the change in treatment rate compared with the preintervention period (0.5 = 50%). the width of the bar represents the number of pregnant women that the clinic saw during the study period. The dotted line represents the mean change across all sites.

During the baseline period, the overall syphilis treatment rate was 70.3% (Table 2). During the nine months after the introduction of the rapid syphilis tests, the syphilis treatment rate increased to 74.7% which was not statistically significant improvement compared to the baseline after adjusting for multiple comparisons (*P* = 0.28). After the 2010 earthquake, the treatment rate returned to 70.2%. Statistically significant improvement in treatment rate was seen after QI to 84.3% compared to each of the previous study periods (*P* < 0.001) Figure 1.

After the rapid test was introduced, treatment rates improved in public clinics (64.4% to 75.3%, *P* = 0.002) but not in private clinics. After the QI intervention, all clinics had improved treatment rates with the greatest gains in private clinics (private: 68.5% to 93.6%, *P* < 0.001; public: 71.6% to 78.1%, *P* = 0.36).

### 3.3. Systems Barriers to Same-Day Testing and Treatment

During the quality improvement intervention, clinics described their key system barriers and the solutions that they used to overcome them. A summary of the systems barriers and solutions is detailed in Table 3.

### 3.4. Adverse Events Averted

Given the geographic spread of this project and the size of the involved clinics, we estimated that if the two interventions were scaled up country wide, this would result in 16,612 additional pregnant women being screened for syphilis, an additional 2,008 pregnant women treated for syphilis, and 567 fewer babies stillborn or born with serious complications of congenital syphilis every year.

## 4. Discussion

Both technology- and health systems-based interventions improve the delivery of prenatal services to prevent congenital syphilis in Haiti. Technology-based solutions in the absence of systems-improvement interventions will have minimal impact on clinical outcomes. In the case of syphilis in Haiti, the availability of a rapid test improved diagnostic testing rates, but it did not significantly affect rates of treatment. The Haitian earthquake in January 2010 did not have significant effects on syphilis testing rates but did cause small but significant declines in syphilis treatment which were overcome during the QI intervention.

Rapid syphilis testing and treatment required a combination of point-of-care testing and systems-based interventions to improve the clinic organization, human resources, supply chain, and other management structures to ensure concurrent improvements to treatment delivery. In a randomized trial from South Africa, point-of-care syphilis testing reduced testing delays; however, there was no significant difference in overall treatment rates [20]. Similar results were found with a point-of-care CD4 test where rapid results had limited impact on HIV treatment initiation [21].

Our results should inform global efforts to eliminate congenital syphilis [22]. Some 10% of women in resource-limited settings are infected with syphilis and over 490,000 babies are born annually with syphilis in Sub-Saharan Africa [23, 24]. In some countries, syphilis is the largest cause of stillbirths and an important cause of premature infant death [25]. Despite availability of rapid tests, prenatal syphilis treatment rates remain below 40% [26]. Our data suggest combining rapid test technology and health systems improvement could lead to large scale implementation success.

Our result are consistent with a growing body of evidence suggesting that quality improvement systems strengthening interventions can successfully increase access to HIV, tuberculosis, and maternal and child health care in resource-limited settings [14, 27–30]. We are now using the QI approach to strengthen HIV and TB integration in Haiti.

These results suggest an important lesson for those introducing new health care technologies in resource-limited settings. When new technologies are introduced, the health system must be adjusted to take advantage of what the new technology offers. This adjustment does not happen automatically without deliberating attention to health system change. In fact, as new therapeutic, diagnostic and preventative technologies are introduced, the gap between what is possible and the actual performance of health systems may grow wider. This “technology to systems gap” means that clinically important discoveries are often held hostage to the inability of our health systems to deliver them. We need parallel investments in systems transformation to ensure that all patients can reap the benefits of clinically meaningful new technologies.

Our data suggest that the introduction of the rapid test technology had its biggest impact in public clinics. We postulate that breakdowns in lab equipment prior to the intervention made rapid-test technology (which require minimal equipment) very advantageous. All clinics, both public and private, benefitted from the quality improvement intervention.

The main strengths of our study were the large sample size and geographic spread across fourteen large clinics in a number of heterogeneous settings. The main limitation of this study is the lack of matched concurrent control facilities. As the study took place within PEPFAR's programmatic constraints, we were not afforded a matched control group. In addition, the high baseline testing rates at all time points did not allow marked improvements in testing rates. While this is certainly a limitation, this does not obscure the impact of the systems improvement intervention on treatment rates.

Our results, despite being encouraging, require further validation in additional implementation research environments, continued study to evaluate sustainability, and costing studies to establish cost-effectiveness of the combined technology and systems-based interventions. Our experience in Haiti demonstrates that health systems improvement efforts coupled with technology can improve care delivery systems to prevent congenital syphilis in resource-poor settings.

## Figures and Tables

**Table 1:** Number and percent of pregnant women tested for syphilis.

	Pregnant women	Tested for syphilis	Percent tested for syphilis
Prerapid test (24 months)	34776	31810	**91.5**
Postrapid test initiation (9 months)	16025	15373	95.9^*^
Prequality improvement (9 months)	14137	13542	**95.8**
Postquality improvement initiation (9 months)	16435	15916	96.8^*^

^*^denotes significant increase compared to prerapid test rates with *P* < 0.001.

**Table 2:** Number and percent of pregnant women treated for syphilis.

	Syphilis positive pregnant women	Treated for syphilis	Percent treated for syphilis
Prerapid test (24 months)	1397	982	**70.3**
Postrapid test initiation (9 months)	652	487	**74.7**
Prequality improvement (9 months)	543	381	**70.2**
Postquality improvement initiation (9 months)	630	531	84.3^*^

^*^denotes significant increase compared to prerapid test rates with *P* < 0.001.

**Table 3:** Health system areas addressed by the intervention, problems, and solutions.

(1) Stock management of syphilis tests and penicillin	
Problems	Solutions
(i) Frequent outages of lab tests and penicillin (ii) No communication within health facility between clinic staff using tests/drugs and stock room/pharmacy (iii) Multiple approvals and administrative layers for facility to reorder rapid syphilis tests and penicillin from national supplier (iv) Requisitions from facility to national supplier often late (v) National supplier often has stock outages of penicillin	(i) Clinic inventory and communication between clinic staff and pharmacy (ii) Earlier requisition by facility of larger stocks from national supplier (iii) Creation of a reserve stock of penicillin (iv) Emergency budget for local procurement of penicillin (v) National supplier of medications includes penicillin as essential drug

(2) Task shifting among health facility staff as syphilis testing moves from laboratory to the point-of-care	
Problems	Solutions

(i) Clinical staff reports insufficient time to perform rapid syphilis tests on every pregnant woman (ii) Laboratory staff reluctant to give up work and fear job loss (iii) Injectable benzathine penicillin located in pharmacy and given by pharmacists only and not in clinic (iv) Job descriptions do not match new responsibilities	(i) Staff becomes more “polyvalent” and performs multiple tasks (ii) Staff backs each other up in case one becomes busy (iii) Laboratory staff comes to clinic to draw blood and perform test on site (iv) HIV counselors taught to draw blood (v) Retraining and continuing education (vi) Team building with goal of preventing newborn death

(3) Patient flow through the health facility	
Problems	Solutions

(i) Multistep process between pregnant woman's arrival to clinic, testing for syphilis, and penicillin injection. “The clinic process resembles an obstacle course for pregnant woman” (ii) Bottlenecks (waiting for clinic chart; waiting for test results; etc.) (iii) Long lines and waits between each step (iv) Frustrated pregnant women leave clinic between steps	(i) Simplify flow and decrease number of steps (ii) Give priority to pregnant women at key points of care (phlebotomy, pharmacy) (iii) “Mobile laboratory staff” within facility to perform lab test in clinic rooms (iv) Group counseling, education, and phlebotomy of all pregnant women in clinic waiting areas (v) Decentralize prenatal care and syphilis screening to satellite facilities to decrease patient volume at large clinics

(4) Data collection and evaluation	
Problems	Solutions

(i) Clinic data flows up to administration, ministry, and PEPFAR but is not available in real time for clinic staff (ii) No locally available indicators on percent of pregnant women tested for syphilis and percent of syphilis positive women treated (iii) Need immediate feedback to reward and motivate staff (iv) Need to correct problems in real time and not wait for a report from a central authority	(i) Use local registers to track a few key indicators (ii) Local “improvement teams” meet regularly to review and report indicators to all staff (iii) Data entry programs redesigned to generate local reports in real time

(5) Patient and community participation	
Problems	Solutions

(i) Women do not know about the dangers of congenital syphilis and importance of screening during pregnancy (ii) Women afraid of injection needle and refuse penicillin (iii) Community needs to hold health facility accountable for syphilis screening	(i) “Bottom up accountability,” by informing women in the community about dangers of congenital syphilis and that they have a right to free prenatal syphilis screening (ii) Community health workers encourage pregnant women to seek prenatal care and screening (iii) Include community members in discussions of how best to provide prenatal care and syphilis screening
